# Notes from Dental Practice

**Published:** 1870-08

**Authors:** James B. Hodgkin

**Affiliations:** Alexandria Va.


### ARTICLE II.

### *Notes from Dental Practice.*

By James B. Hodgkin, D. D. S. Alexandria Va.

### *Health Restored by the Extraction of Diseased Teeth.*

Mrs. R. aged 38 called to see about her teeth. She had been suffering for a long time with distressing pains in her head, which had been so great a torment to her as to render her life a burden. Her sight was much affected, the eyes being red and watery, and her hearing also was impaired. On examination all the upper teeth were found decayed so badly as to permit no hope of their restoration, most of them being mere stumps; she stated that they had not given her any particular trouble and that her physician had told her she had better have them extracted, this was done with all the stumps save two, which from the difficulty attending their removal and her sensitiveness were left remaining. She was requested to call in about three weeks when they would probably be in a better condition and her nerves more firm. She called at the time appointed and expressed herself much gratified with the results of the operation she had submitted to, as her troubles were much mitigated, hearing and sight improved, and the distressing pain in her head almost gone. The remaining roots were now removed, and she decided to wait until she could have a permanent set of teeth before having the plate constructed. At the appointed time Mrs. R. called to have the artificial teeth constructed, and stated that the restoration of her health was entire, and has been since, a period of two years.

---

*Iron Retorts for Nitrous Oxide.*

The fifty gallon gasometer sent me from the manufactory of Snowden & Cowman, a year or more ago has answered my purpose excellently well, as did also the glass retort accompanying it—until it broke, which was not long. After exhausting the supply of a firm here, at the rate of about one a week, as all the retorts not in the private use of druggists and others were used up, I concluded that if a more substantial material would answer I would try and obtain an unbreakable retort, and accordingly ordered a 20 ounce iron one as a sort of experiment. I am proud to say that after several months use of this latter material I think it quite as good as glass; a little less convenient perhaps, as one has to graduate the heat by the judgement, not being able to see the condition of the contents of the retort, but with a little practice this and other difficulties are overcome, and a very excellent article of gas is made without the constant dread of breakage formerly felt. I dont try to have "the gas fresh every day" however, as I find that when I can keep it a month it is pleasanter and fully as efficacious, while that six weeks old is still better. I was not a little amused a short time ago, at the faith of a lady in a Chicago dentist's statement, made to her while visiting that enterprising city, to the effect that she must never under any consideration allow a dentist to give her any other gas than that made the day before being taken, or if possible made the same morning it was inhaled, as all gas not perfectly fresh was highly poisonous. Finding her faith well founded in Chicago "gas" I let her use, wondering to myself if the world ever would grow wise enough to take down the sign hung at every door: "Premiums awarded to humbuggery."

To return to the retort question, if some manufacturers would make a flask shaped retort, porcelain lined, it would sell well I am sure. I find the inch gas-pipe which conveys away the gas from the retort to the wash-bottles is corroded quite rapidly by the action of the decomposed salt. A retort shaped like an old-fashioned glass one, with a short neck and porcelain lined would be just the article needed.

---

### ARTICLE III.

### *The Death of Dr. W. L. Bowdoin.*

